# Comparing and assessing physical activity guidelines for children and adolescents: a systematic literature review and analysis

**DOI:** 10.1186/s12966-020-0914-2

**Published:** 2020-02-10

**Authors:** Anne-Maree Parrish, Mark S. Tremblay, Stephanie Carson, Sanne L. C. Veldman, Dylan Cliff, Stewart Vella, Kar Hau Chong, Maria Nacher, Borja del Pozo Cruz, Yvonne Ellis, Salome Aubert, Billie Spaven, Mohd Jamil Sameeha, Zhuiguang Zhang, Anthony D. Okely

**Affiliations:** 1 Faculty of Social Sciences, University of Wollongong, Wollongong, NSW 2521 Australia; 2 Early Start, University of Wollongong, Wollongong, Australia; 3 Illawarra Health and Medical Research Institute, University of Wollongong, Wollongong, Australia; 4 Healthy Active Living and Obesity Research Group, Children’s Hospital of Eastern Ontario Research Institute, Ottawa, Canada; 5 Department of Public and Occupational Health, Amsterdam Public Health Research Institute, Amsterdam University Medical Center, Amsterdam UMC, Vrije Universiteit Amsterdam, Amsterdam, The Netherlands; 6 Motivation and Behavior Program, Institute for Positive Psychology and Education, Faculty of Health Sciences, Australian Catholic University, Sydney, Australia; 7 Nutritional Science Programme, Centre for Community Health, Faculty of Health Sciences, Universiti Kebangsaan Malaysia, Kuala Lumpur, Malaysia

**Keywords:** Recommendation, Guideline, Physical activity, Sedentary behaviour, Movement, Children, Youth, Adolescents, AGREE II, Grey literature

## Abstract

**Background:**

The impact of declining physical activity and increased sedentary behaviour in children and adolescents globally prompted the development of national and international physical activity guidelines. This research aims to systematically identify and compare national and international physical activity guidelines for children and adolescents and appraise the quality of the guidelines to promote best practice in guideline development.

**Methods:**

This systematic review was registered in the International Prospective Register of Systematic Reviews (PROSPERO) and reported using the Preferred Reporting Items for Systematic Reviews and Meta-Analysis (PRISMA) guidelines. Only national, or international physical activity and/or sedentary behaviour guidelines were included in the review. Included guidelines targeted children and adolescents aged between 5 and 18 years. A grey literature search was undertaken incorporating electronic databases, custom Google search engines, targeted websites and international expert consultation. Guideline quality was assessed using the Appraisal of Guidelines for Research and Evaluation II Instrument (AGREE II).

**Results:**

The search resulted in 50 national or international guidelines being identified. Twenty-five countries had a national guideline and there were three international guidelines (European Union, Nordic countries (used by Iceland, Norway and Sweden), World Health Organization (WHO)). Nineteen countries and the European Union adopted the WHO guidelines. Guidelines varied in relation to date of release (2008 to 2019), targeted age group, and guideline wording regarding: type, amount, duration, intensity, frequency and total amount of physical activity. Twenty-two countries included sedentary behaviour within the guidelines and three included sleep. Total scores for all domains of the AGREE II assessment for each guideline indicated considerable variability in guideline quality ranging from 25.8 to 95.3%, with similar variability in the six individual domains. Rigorous guideline development is essential to ensure appropriate guidance for population level initiatives.

**Conclusions:**

This review revealed considerable variability between national/international physical activity guideline quality, development and recommendations, highlighting the need for rigorous and transparent guideline development methodologies to ensure appropriate guidance for population-based approaches. Where countries do not have the resources to ensure this level of quality, the adoption or adolopment (framework to review and update guidelines) of the WHO guidelines or guidelines of similar quality is recommended.

**Trial registration:**

Review registration: PROSPERO 2017 CRD42017072558.

## Background

A growing body of evidence demonstrates the relationship between physical activity and positive health outcomes in children and adolescents [1], whilst excessive time spent in sedentary behaviours, and particularly screen time, is negatively associated with health outcomes [2, 3]. Over the past three decades global concerns regarding declining levels of physical activity and the subsequent impact on health outcomes prompted several national and international governing bodies to develop guidelines providing recommendations for policy makers, practitioners and individuals [4, 5]. Early iterations of physical activity guidelines for children were based on adult recommendations [5]. In 1994 the United States (US), was the first country to produce physical activity guidelines specifically customized for adolescents [6], which were later followed by guidelines for ‘school aged youth’ in 2004 [7]. Over this period of time, the United Kingdom, Canada and Australia released guidelines for children and youth [8]. In the past decade there has been a trend encouraging a more transparent and rigorous approach [9] to guideline development with growing bodies of evidence and more recent guideline development frameworks Appraisal of Guidelines for Research and Evaluation II Instrument (AGREE II) [10]. Canada released the world’s first stand-alone sedentary behaviour guidelines for children and youth in 2011 [11]. More recently national and international bodies have included recommendations for sedentary behaviour in their physical activity guidelines due to the growing body of evidence linking excessive sedentary behaviour to poor health outcomes [2, 3]. Much of this evidence centred on screen-based sedentary pastimes [2, 3]. In 2016, Canada became the first country to replace their national physical activity and sedentary behaviour guidelines for children and adolescents with 24-h movement guidelines, which consider behaviours across a ‘24-hour movement spectrum’ and also included recommendations for sleep [12]. New Zealand adopted the Canadian guidelines in 2017 and Australia used the Grading of Recommendations Assessment Development and Evaluation (GRADE) recommended GRADE-ADOLOPMENT approach to develop 24-h movement guidelines from the Canadian guidelines in 2019 [13]. This approach is a structured, transparent, cost effective process to review and update guidelines based on an evidence-to-decision framework using previous guideline systematic reviews which are updated to reflect the date of guideline development.

As the evidence-base supporting guideline development continues to grow, more countries have implemented guidelines to inform parents, health professionals and policy-makers of recommended levels of physical activity for children and adolescents [14]. In the past 5 years, numerous countries have reviewed or updated their physical activity guidelines for children and adolescents, with a trend towards more robust evidence-based guidelines. Cross-country comparisons of guidelines revealed variability in age categories, activity duration, intensity, frequency, type of activity/sedentary behaviour and overall guideline quality [14]. With escalating rates of non-communicable disease globally, prevention is imperative; evidence-based, high quality physical activity guidelines are essential to guide practitioners, professionals, policy makers and the public, and avoid confusion and misinterpretation of the underlying evidence-base. The purpose of this systematic review was to identify national and international organizations with existing official physical activity and/or sedentary behaviour guidelines for school-aged children and adolescents (5–18 years), appraise the quality of the guidelines, draw comparisons between the guidelines, and recommend standards to promote best practice and opportunities for cross-country comparisons.

## Methods

### Design

This systematic review was registered with the International Prospective Register of Systematic Reviews (PROSPERO; Registration no CRD42017072558) [15]. It is reported using the Preferred Reporting Items for Systematic Reviews and Meta-Analysis (PRISMA) statement for reporting systematic reviews and meta-analyses [16].

### Information sources and search strategies

The search strategies for this review were developed during the meeting of co-investigators (AMP, TO, DC, SV, MT). Two research librarians then provided expert advice to further develop and refine the strategy. As most documentation for the review is not commonly found through scholarly literature sources, it was determined that the most appropriate methodology would be to use a grey literature search plan [17]. This strategy was adapted from a previous review that used grey literature search methods to examine guidelines for breakfast programs in Canada [17].

For a guideline to be included in this review it had to incorporate a statement from a national or international institution outlining the physical activity and/or sedentary behaviour recommendations for children and adolescents between the ages of 5 and 18 years, as defined in the review eligibility criteria in Table 1 [17]. There were no language restrictions. Records included peer-reviewed journals and grey literature sources of guideline documents or webpages published between January 2010 (the date that the World Health Organisation released the first international guidelines) [18] and the date of the searches. Key search terms included: “physical activity”, “exercise”; “guideline*”, “recommendation*”; “child*”, “youth”, “adolescen*”, “school aged”, “young pe*”, “child*”.

**Table 1:** Review eligibility criteria

Inclusion criteria	Exclusion criteria
Published by Government or Non-Government Organization at the Federal/National level	Document is a draft version or has been replaced with another document
No language restrictions	Newsletters, news releases or memoranda
Most current version of the document	
The document must incorporate a statement outlining the physical activity and/or sedentary behavior guidelines or recommendations for children and youth/adolescents between the ages of 5 and 18 years	

The grey literature search involved four search strategies: (1) grey literature databases, (2) custom Google search engines, (3) targeted websites, and (4) consultation with content experts [17]. The grey literature database search included PubMed, ProQuest and CINAHL databases. These databases were selected upon consultation with the University research librarians and were deemed appropriate due to their ability to include grey literature. The search of these databases commenced on the 18th July 2017 (BS) and concluded on the 20th of July 2017 (BS) (see Additional files 1, 2 and 3 for more detail). Records identified in this search were extracted from the online interfaces and imported into EndNote referencing software [19] The search was re-run and updates made on the 7th March 2019 (AMP and SLCV).

The Google search included Google and Google Scholar. This search was limited to and included sources from 2010 when the last iteration of the World Health Organisation (WHO) were released until March 2019. Google searches yield an overwhelming number of results, due to the fact that Google search engines use relevancy ranking bringing the most relevant sources to the top of the search results. In keeping with previous research [17], the first 15 pages (150) results were included in the review. In addition, pages 16 and 17 of the Google search results were manually checked to ensure the relevancy of this method. These results were tagged and imported into Zotero software [20] and then transferred into the EndNote referencing software [19].

The third search included targeted websites of government and health organizations. The first targeted web search occurred on the 18th of July 2017 and was updated in March 2019. This search included the following sources: the WHO website, EuroScan International Information Network, International Network of Agencies for Health Technology Assessment (INAHTA), OpenGrey and WorldWideScience. In addition, the following limiters were used in the Google search engine: site:org and/or site:gov. Records identified in this search were extracted from the online interfaces into Zotero [20] software and then transferred into the EndNote software [19].

The final search strategy involved contacting content experts to seek their recommendations for document inclusion in the review. The Active Healthy Kids Global Alliance organised a Global Matrix on physical activity for children and adolescents, involving leading international experts from 49 countries who participated in the preparation of national physical activity report cards for children and adolescents [21]. Designed to raise awareness of physical activity participation levels, the report cards assign grades to physical activity indicators based on country specific data. These experts were contacted and surveyed in March 2019 to identify which report card they had led, which physical activity and/or sedentary behaviour guidelines they followed, and the associated links to guideline documents (Additional file 3). Identified guidelines and associated documentation were manually entered into the EndNote software [19].

**Fig. 1:**
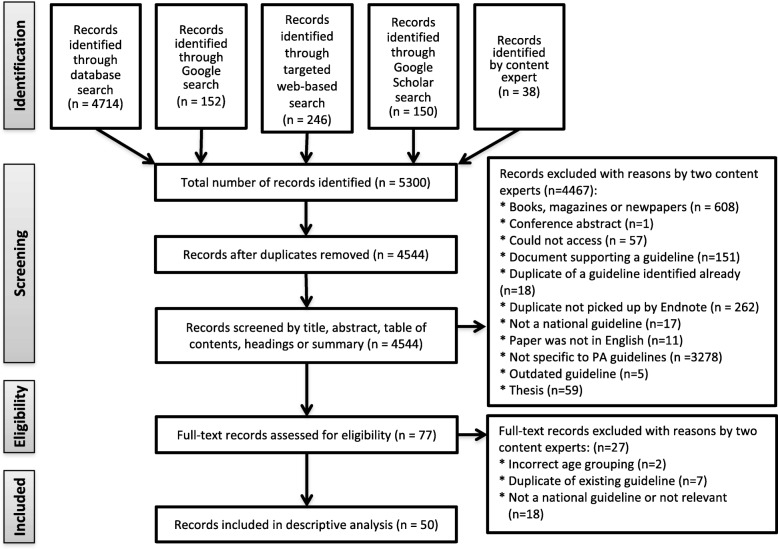
PRISMA flow diagram of study selection

Once identified records were all entered into the Endnote software, de-duplication took place prior to proceeding to level one screening and all duplicates were removed as were books, magazines and newspapers (Fig. 1). Level one screening included independent screening of relevant titles and abstracts, webpages and guideline documents by the two reviewers (BS and AMP; SLCV and AMP update). Any document included by one reviewer and not the other was retained for further review at level two. Level two involved examination of potentially eligible full text documents or webpages that were retrieved and independently assessed for eligibility by the two review team members (BS and AMP; SLCV and AMP update). The reference list of relevant review papers was manually checked for papers potentially missed by the search. The homepage of relevant webpages was searched for potentially relevant documents. Disagreements regarding eligibility of guideline documents were resolved through discussion with a third reviewer (ADO or DC).

**Fig. 2:**
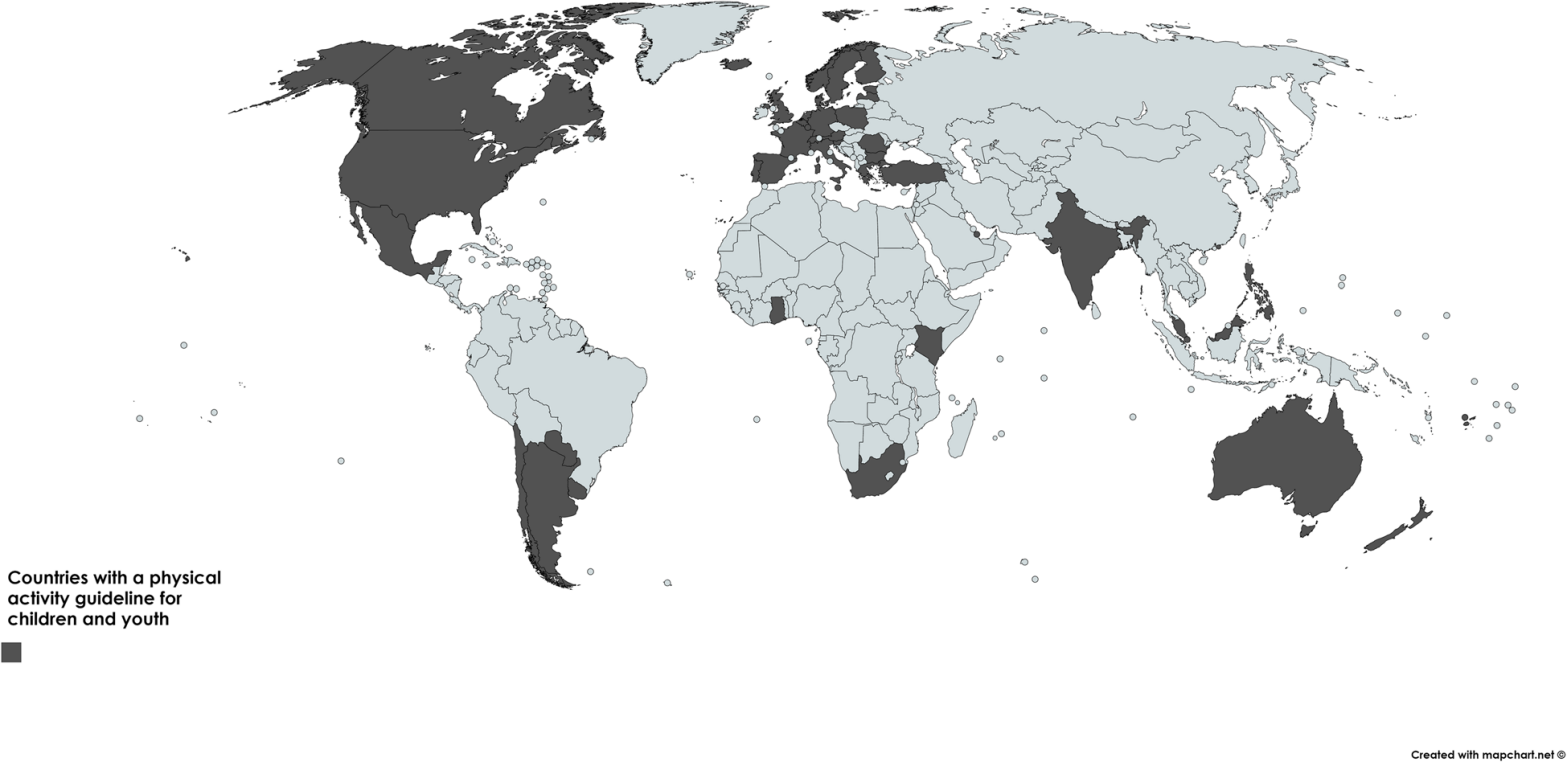
Map of countries with guidelines

A standardised, pre-piloted form was used to extract data from the included documents to allow assessment of quality and evidence synthesis. The form included: country, name of guideline, issuing authority, date of release, age group, recommended physical activity duration, intensity, frequency, type and sedentary behaviour recommendation (Table 2). Data extraction was completed by one reviewer (AMP) and verified by another reviewer (DC).

**Table 2:** Detailed guideline summary

Descriptors				Recommendations							Reference
Country	Issuing authority	Date of release	Age group	Duration	Intensity	Frequency	Inclusion of vigorous physical activity	Additional information	Bouts	Inclusion of a sedentary behaviour recommendation	Guideline link or pdf
Argentina *Manual Director De Actividad Física Y Salud De La Republica Argentina*	Ministerio De Salud De La Nación (Spanish)	2013	5–17 years	At least 60 min	Moderate to vigorous	Every Day	Should incorporate intense activities at least 3 times per week.	Physical activity > 60 min reported additional health benefits. Daily physical activity should be, for the most part, cardiorespiratory endurance. Should include activities to strengthen muscles and bones, at least 3 times a week			https://www.slideshare.net/GESAD/manual-director-de-actividad-fsica-de-la-repblica-argentina
Australia	Australian Government Department of Health	2019	5–17 years	At least 60 min	Moderate to vigorous (involving mainly aerobic activities)	Every day	Activities that are vigorous at least 3 days per week.	Several hours of a variety of light physical activities. As well as those that strengthen muscle and bone should be incorporated at least 3 days per week. Sleep: An uninterrupted 9 to 11 h of sleep per night for 5–13 years and 8 to 10 h per night for 14–17 years. Consistent bed and wake-up times.		Limiting sedentary recreational screen time to no more than 2 h per day. Breaking up long periods of sitting as often as possible. For greater benefit replace sedentary time with additional MVPA, while preserving sufficient sleep.	http://www.health.gov.au/internet/main/publishing.nsf/Content/health-24-hours-phys-act-guidelines
Austria *Austrian recommendations* *For health-enhancing physical Activity*	Federal Ministry of Health, Health Austria GmbH and Business Unit Fund Healthy Austria	2013	School aged children and adolescents	At least 60 min	Moderate to vigorous	Every day	Several times a week activities that stimulate the cardiovascular system through endurance sport activities.	Several times a week activities that build strong bones, strengthen muscles, improve agility and maintain flexibility Several times a week: activities that improve agility and maintain flexibility Children at primary school should engage in considerably more physical activity.		Avoid long periods of inactivity- It is recommended that individuals avoid long periods of physical inertia as much as possible or that they punctuate such periods lasting around two hours or longer with active stints of physical activity.	http://fgoe.org/sites/fgoe.org/files/2017-10/2012-10-17.pdf
Canada	Canadian Society for Exercise Physiology (CSEP)	2016	5–17 years	Accumulate at least 60 min	High levels of physical activity, low levels of sedentary behaviour, and sufficient sleep.	Every day	Vigorous PA should each be incorporated at least 3 days per week.	Several hours of a variety of structured and unstructured light physical activities Muscle and bone strengthening activities should each be incorporated at least 3 days per week. Sleep: Uninterrupted 9 to 11 h of sleep per night for 5–13 years and 8 to 10 h per night for 14–17 years.		Sedentary behaviour: no more than 2 h per day of screen time and limit sitting for extended periods.	https://indd.adobe.com/view/b82b4a90-6e46-4b1a-b628-d53805688baf
Chile *Recomendaciones para la práctica de Actividad Fisica segun curso de vida*	Ministerio de Deporte, Saludy Educacion (Spanish)	2017	0–3 years 4–6 years 7–9 years 10–17 years	At least 60 to 90 min	Moderate to vigorous	Every day		Muscle and bone strengthening activities should be incorporated 2 or more days per week. Activities for muscle strengthening and flexibility at least 2 times per week. Encourage active transport and outdoor physical activities. Promoto to be active at home, at the school and in general during daily routine.	Aerobic activities can be done in bouts of at least 10 min.		http://www.mindep.cl/wp-content/uploads/2016/06/Recomendaci%C3%B3n-para-la-pr%C3%A1ctica-de-actividad-f%C3%ADsica-seg%C3%BAn-curso-de-vida.pdf
China	National Children’s Medical Center	2018	6–17 years	Minimum of 60 min	Moderate to vigorous (involving mainly aerobic activities)	Every day	Recommend vigorous PA be incorporated at least 3 days per week.	Recommend bone and muscle strengthening activities be incorporated at least 3 days per week.		Limiting screen time to no more than 2 h per day. Reducing prolonged sedentary behaviour at class. Promoting physical activity during break times.	张云婷, et al. “中国儿童青少年身体活动指南.” *中国循证儿科杂志* 12.6 (2017): 401–409.
Finland	National Institute for Health and Welfare	2008 for PA 2015 for SB	7–18 years	At least 1 to 2 h	Physically active	Every day		In a variety of ways suitable for each age group. At least two hours for a 7-year-old and at least an hour for an 18-year-old	The daily dose of exercise should include several at least 10 min of brisk exercise periods	Do not sit still continuously for longer than one hour. Sitting still for more than two hours straight should be avoided. Screen time with entertainment media should not exceed two hours a day.	https://julkaisut.valtioneuvosto.fi/bitstream/handle/10024/69943/978-952-00-3417-7_korj.pdf?sequence=1&isAllowed=y http://julkaisut.valtioneuvosto.fi/handle/10024/74710
France	French Agency for food environmental and occupational health safety	Updated 2016	6–11 year and 12–17 years	At least 60 min (6–11 years) 60 min (12–17 years)	MVPA	Every day		6-11 years: 1 h of MVPA daily 12-17 years: 1 h of MVPA daily exercising the muscles and improving stamina and flexibility Sleep: 6–11 years 9–11 h of sleep 12–17 years: 8.5–9.5 h of sleep	At least 3 sessions of at least 20 min of high intensity PA (non-consecutive days)	6–11 years: no more than 2 h per day in front of a screen 12–17 years: Limit time in front of a screen and avoid staying in a sitting position for more than 2 consecutive hours	https://www.anses.fr/en/content/physical-activities-%E2%80%93-our-recommendations-children-and-adolescents
Germany	German Federal Ministry for Health	2016	6-11 years and 12-18 years	90 min or more; (60 min on every day activities e.g. at least 12,000 steps)	MVPA	Every day		For primary school aged children, the large muscle groups should be subject to higher-intensity loading on two to three days a week to improve strength and endurance. Physically inactive children and adolescents should be introduced gradually to the target, e.g. initially 30 min of physical activity on one to two days per week. The duration is then increased first, after which the intensity is increased		Reduce avoidable sitting to a minimum; particularly reduce screen media to a minimum. Primary school children maximum 60 min/day Adolescents: maximum of 120 min/day	https://www.sport.fau.de/files/2015/05/National-Recommendations-for-Physical-Activity-and-Physical-Activity-Promotion.pdf
Ghana	Ministry of Health	2009	Children and adolescents (age not stated)		Moderate to vigorous	Every day	Vigorous aerobic exercise at least 3 days a week.	Bone-strengthening physical activity 3 days a week.			http://alwag.org/education/courses/pa-guide.pdf
Malaysia	Malaysian Ministry of Health	2017	5–17 years	At least 60 min	Moderate or vigorous (involving mainly aerobic activities)	Every day	Vigorous intensity should be incorporated	Amounts of physical activity greater than 60 min provide additional health benefits. Activities that strengthen muscle and bone such as squat, push-ups, curl-ups and lunges should be incorporated. It is recommended that 30 min of physical activities should be performed in the morning and the rest in the evening depending on the individual’s schedule.			https://mdes.org.my/wp-content/uploads/2017/07/garis-panduan-aktiviti-fizikal-2017.pdf
Mexico	National council for science and technology and Board of Directors of the National Academy of Medicine in agreement with the WHO	2015	5–17 years	Should accumulate at least 60 min	Moderate or vigorous or a combination from both.	Every day	Vigorous –intensity should be incorporated	Including those that strengthen muscle and bone at least 3 times per week. Increase physical activity for more than 60 min a day has additional health benefits. Daily physical activity should be mostly aerobic, such as walking, running, jumping, dancing.	It can consist of several session throughout the day (e.g. two runs of 30 min)		http://guiasalimentacionyactividadfisica.org.mx/wp-content/uploads/2015/10/Guias-alimentarias-y-de-actividad-fisica.pdf
Netherlands	The Health Council Netherlands	2017	4–18 years	At least 60 min	Moderate to vigorous	Moderate intensity: every day	Heavy intensity: at least 3 times per week	Include activities that strengthen muscle and bone at least 3 times per week.		Avoid spending long periods sitting	https://www.healthcouncil.nl/documents/advisory-reports/2017/08/22/physical-activity-guidelines-2017
New Zealand	Ministry of Health New Zealand	2017	5–17 years	At least 60 min	Moderate or vigorous	Every day	Include vigorous activity at least 3 days a week.	Activities that strengthen muscles and bone at least 3 days a week. Include a variety of light physical activity for several hours a day. Get enough sleep 5–13 year olds, get 9–11 h of quality uninterrupted sleep each night. 14–17 year olds, get 8–10 h of quality uninterrupted sleep each night. Have a regular bed time and wake up time.		Don’t spend much time sitting no more than 2 h a day on recreational screen time. Sit less move more and break up sitting.	https://www.health.govt.nz/your-health/healthy-living/food-activity-and-sleep/physical-activity/how-much-activity-recommended
The Nordic Nutrition Recommendations (NNR)	Nordic Council of Ministers	2012	Children and adolescents (age not specified)	At least 60 min	Moderate to vigorous	Every day	Vigorous activity should be incorporated at least 3 times per week.	Incorporate activities that strengthen muscle and bone at least 3 times/week Physical activity of amounts greater than 60 mins daily will provide additional health benefits. Activities should be as diverse as possible in order to provide optimal opportunities for developing all aspects of physical fitness, including cardio-respiratory fitness, muscle strength, flexibility, speed, mobility, reaction time, and coordination.		Reduce sedentary behaviour	http://norden.diva-portal.org/smash/get/diva2:704251/FULLTEXT01.pdf
Paraguay	Directorate of surveillance of non-transmissible diseases; General directorate of health surveillance	2014	5–17 years	Accumulate at least 60 mins	Moderate to vigorous	Every day	Vigorous physical activity 3 times per week	Activity to strengthen muscles and bones 3 times/week. Physical activity for more than 60 mins/day brings additional benefits for health. Daily PA should be for the most part cardio-respiratory resistant (aerobic).	The period of 60 mins/day can be done in several sessions throughout the day e.g. 2 run blocks of 30 mins		http://www.vigisalud.gov.py/documentos/01_07_2016_15_47_12_Manual-de-Actividad-Fisica.pdf
Philippines	Philippine Department of Health	2010	5–12 years and 13–20/21 years	60 min	Physical activity broken into (Active daily tasks, Exercise dance or sports and High impact play)	Every day	5–12 years: Include high impact unstructured play (e.g. running, jumping) 13–20 years: Include high impact unstructured play at least 20 mins of sustained MVPA (brisk walking or jogging) for a minimum of 30 mins	5–12 years: programed PA for 20–30 min each day (sports or active games). Include high impact unstructured play (e.g. running, jumping) 13–20 years at least 40 mins of programmed PA (fitness related rhythmic or sports activities). For fitness goals you should have 20–30 min minimum for at least 3–5 times/week. At least 2–3 times a week of activities that build muscle and bone strength and flexibility such as weight bearing calisthenics and other load bearing exercises involving major muscle groups.	13–20 years at least 20 mins of sustained MVPA continuously for a minimum of 30 mins OR accumulated bouts of 10 mins or longer		https://www.doh.gov.ph/sites/default/files/publications/HBEAT58a.pdf
Qatar	State of Qatar National Physical activity guidelines	2014	5–11 years and 12–17 years	At least 60 min	Moderate to vigorous	Every day	Vigorous activity at least 3 times/week	Strength training at least 3 times per week		Reduce sitting time. Reduce time spent in front of electronic devices. Take an activity break every hour of sitting Reduce sitting time. Limit screen time to less than 2 h. Take an activity break every hour of sitting	https://www.namat.qa/NamatImages/Publications/75/QATAR%20PA%20GUIDLINE%20ENGLISH.PDF
Singapore	Singapore Health Promotion board	2011	7–18 years	60 min	MVPA	Everyday	Incorporate vigorous intensity physical activity on at least 3 times per week as part of the 60 min	Incorporate physical activity that strengthen muscle and bones at least 3 times per week as part of the 60 min		Limit total sedentary entertainment screen time (e.g. TV & video games) to < 2 h/day. Break up sedentary period (except time spent sleeping) lasting longer than 90 mins with 5–10 min of standing, moving around, active play or doing some PA	https://www.academia.edu/10443994/National_Physical_Activity_Guidelines_for_Children_and_Youth
South Africa	Department of Health Republic of South Africa	2013	5–17 years	At least 60 min	MVPA	Every day		Activities that strengthen the muscles and bones of children should be performed three times a week			http://www.fao.org/3/a-as842e.pdf
Spain	Ministry of Health, Social Services and Equality	2015	5–17 years	At least 60 min	Moderate to vigorous	Every day	Include at least 3 days/week vigorous activities	Include at least 3 days/week activities that strengthen muscle and improve bone mass. Muscle strengthening and bone mass improvement activities that include large muscle groups. Moderate/vigorous intensity aerobic activity. Encourage active transport and outdoor activities		Reduce prolonged sedentary periods. Limit screen time to a maximum of 2 h a day.	https://www.mscbs.gob.es/profesionales/saludPublica/prevPromocion/Estrategia/docs/Recomendaciones_ActivFisica_para_la_Salud.pdf
Switzerland	Federal Office of Sport & Federal Office of Public Health	2012	School age children and adolescents	At least 60 min	Moderate to vigorous	Everyday		Undertake a varied range of physical activities and sports: • Build strong bones through weight-bearing and strength-building activities • Stimulate the cardiovascular system • Strengthen muscles • Improve agility (coordination) • Maintain flexibility		Avoid long periods of inactivity	https://www.hepa.ch/de/dokumentation.detail.document.html/hepa-internet/de/documents/en/bewegungsempfehlungen/hepa_Gesundheitswirksame%20Bewegung_Grundlagendok_EN.pdf.html
Turkey	Republic of Turkey; Ministry of Health- Public Health Institution	2014	5–17 years	60 min	MVPA	Every day	Vigorous intensity at least 3 times/week	Activity for more than 60 mins provides extra benefits. Endurance activities (strengthening) are recommended. Shorter activities provide benefits for inactive children. Adolescents aged 12–18 years accumulate 60 min of MVPA; should include VPA 3 times per week and muscle and bone strengthening activities 3 days per week. A well planned PA program should include 4 activities: endurance (aerobic), muscle and bone strengthening, weight lifting, balance and stretching activities.	Activities can be performed in multiple shorter periods spread throughout the day.	Not recommended for children to stay sedentary for a long period of time.	http://beslenme.gov.tr/content/files/basin_materyal/Fiziksel_aktivite_rehberi/ingilizce.pdf page 9
United Kingdom (UK)	Department of Health and Social Care UK	July 2011	5–18 years	At least 60 min and up to several hours	Moderate to vigorous	Every day	Vigorous intensity physical activity should be incorporated at least 3 days/week	Activity that strengthens muscle and bone, should be incorporated at least 3 days/week		All children should minimise the amount of time spent being sedentary for extended periods	https://www.gov.uk/government/publications/uk-physical-activity-guidelines
United States (USA)	U.S. Department of Health and Human Services (HHS)	2018	6–17 years	60 min or more	Moderate to vigorous activity	Every day	Vigorous intensity at least 3 days of the week	As part of the 60 min or more of daily physical activity, children and adolescents should include muscle strengthening physical activity on 3 days a week. As part of the 60 min or more of daily physical activity, children and adolescents should include bone-strengthening physical activity on at least 3 days a week.			https://health.gov/paguidelines/second-edition/pdf/Physical_Activity_Guidelines_2nd_edition.pdf (page 46)
Uruguay	Ministry of Public Health & National Secretariat of Sport Ministry of Health	Unknown (note document cites a reference from 2016)	5 years to pre-pubertal and Adolescents	At least 60 min/day	Moderate to vigorous	Every day		Include exercises that help strengthen muscle and bones Incorporate strength exercises at least twice a week		Decrease the times of sitting, especially in front of the screens or cell phones and televisions.	https://www.gub.uy/ministerio-salud-publica/comunicacion/publicaciones/guia-actividad-fisica
World Health Organisation (WHO)	WHO	2010	5–17 years	At least 60 min	Moderate to vigorous	Every day	Vigorous –intensity should be incorporated at least 3 times per week.	Amounts of physical activity > 60 min provide additional health benefits. Most daily physical activity should be aerobic. Including those that strengthen muscle and bone at least 3 times per week.			http://www.who.int/dietphysicalactivity/publications/9789241599979/en/

Re: Belgium, Bulgaria, Denmark, Estonia, European Union, Fiji, Greece, Hong Kong, India, Italy, Kenya, Latvia, Lithuania, Luxembourg, Malta, Poland, Portugal, Romania, Slovakia, Slovenia, use the international WHO guidelines for physical activity. Iceland, Norway and Sweden use the Nordic Nutrition guidelines

If a document stated that a country used more than one guideline to create their country’s guideline (e.g., WHO and Centre for Disease Control (CDC)), the guideline was included in Table 2. In some instances, experts indicated that their country had a national physical activity guideline; however if there was no documented evidence to support this claim it was not included. In other instances, experts stated that the country’s guideline was based on either the WHO, CDC or Canada’s physical activity guideline, however, if this could not be verified with documented evidence, these countries guidelines were not included.

### Guideline quality

The quality of each national and international guideline was assessed using AGREE II. The original instrument was developed in 2010 and was updated in 2017 [10]. It includes six categories and 23 items with 7-point Likert scales. The AGREE II instrument is a valid and reliable instrument for assessing guideline quality [22, 23]. Assessors used the AGREE II Instrument manual and on-line training tool [10]. Two people independently assessed each guideline. Ten assessors (AMP, SC, KHC, MNE, BdPC, SA, MJS, CT, YE, ZZ) were involved in appraising the guidelines using the AGREE II instrument due to the variation in languages. As per AGREE II instrument guidelines, quality scores are calculated for each of the six domains by ‘summing all the scores for each of the individual items in a domain and scaling the total as a percentage of the maximum possible score for that domain’ [10]. Guidelines from 27 countries were evaluated by at least two appraisers. In instances where the two assessors’ evaluation of AGREE II items varied by a margin of more than two points, assessors revisited the item to find a consensus to reduce the gap in the margin of their assessment. In four instances a third assessor was consulted to assist in this process, due to the unavailability of the original reviewer.

## Results

### Countries with guidelines

The search resulted in the identification of 50 verified national or international guidelines on physical activity and/or sedentary behaviour for children and adolescents (Table 2 and Fig. 2). A quick summary of the guidelines can be found in Table 3. Twenty-five countries had national guidelines. There were three international guidelines including the European Union [24] (which follows the WHO guideline), the Nordic [25] (Iceland, Norway and Sweden used these guidelines) and the WHO guidelines [18]. The WHO guidelines were adopted by 19 other countries and by the European Union. No countries made specific reference to the European Union guidelines. Countries that based guidelines on the WHO physical activity guidelines [18] or the Nordic Nutrition guidelines [25] are mentioned at the bottom of Table 2. For three national South Africa [26]; Estonia [27]; Kenya [28] and one international guideline (Nordic) [25], the physical activity guidelines were incorporated into nutrition/dietary guidelines. Venezuela and South Korea were believed to have a national physical activity guideline, however a guideline could not be found. Croatia, Cyprus and the Czech Republic had customized WHO country factsheets; however, the factsheets stated that they did not have a national guideline and that it was under development. Some national guidelines were identified as following either the WHO [18], Canadian [29] or the United States [30] physical activity guidelines, yet no documented evidence could be found; these countries included: Brazil (WHO), Columbia (WHO), Mozambique (Canadian), Nigeria (Canadian), Thailand (WHO), United Arab Emirates (WHO and United States), and Zimbabwe (WHO).

### Guideline content

#### Date of guideline release and age category

The date of release of the guidelines ranged from 2008 to 2019. There was considerable variability between the age categories specified in the guidelines for children and adolescents (refer to Table 2). Age categories for children and adolescent guidelines ranged from 0 to 21 years of age. The most common category was 5–17 years 12 countries/international guidelines used this age category including: Argentina [31], Australia [32], Canada [29], Malaysia [33], Mexico [34], New Zealand [35], Paraguay [36], South Africa [26], Spain [37], Turkey [38], WHO [18] and Qatar [39] (Qatar also had sub categories of 5–12 years and 12–17 years). Further details of variation in this category can be found in Table 2.

#### Physical activity duration

More homogeneity existed between guidelines in reference to ‘time spent’ being physically active. All except one country (Germany [40]) indicated that children should participate in 60 min of physical activity daily; however there was variability in the wording of the recommendations. Germany recommended 90 min or more (“60 minutes on every day activities e.g., at least 12,000 steps”). More detail regarding slight wording variations can be found in Table 2.

#### Physical activity intensity

The majority of countries [19] recommended children’s daily physical activity consist of moderate to vigorous physical activity (MVPA) (Argentina [31], Austria [41], Chile [42], France [43], Germany [40], Ghana [44], New Zealand [35], Nordic [25], Paraguay [36], Qatar [39], Singapore [45], South Africa [26], Spain [37], Switzerland [46], Turkey [38], United Kingdom [47], United States [48], Uruguay [49], WHO [18]). The remaining countries used slight variations in the wording (refer to Table 2).

Twenty countries guidelines referred to vigorous physical activity (VPA). Seven indicated VPA should be engaged in at least 3 times per week (Argentina [31], New Zealand [35], Nordic [25], Paraguay [36], Singapore [45], Turkey [38], WHO [18]), while seven other countries recommended VPA for 3 days per week (Australia [32], Canada [29], China [50], Ghana [44], Spain [37], United Kingdom [47], United States [48]). The remaining countries used slight wording variations which can be found in Table 2, however the Philippine guidelines contained some ambiguity: ‘for 5-12 years include high impact unstructured play (e.g. running, jumping) and for 13-20 years include ‘high impact unstructured play at least 20 mins of sustained MVPA (brisk walking or jogging) for minimum of 30 mins’ (Philippines) [51].

#### Muscle and bone strength

Twenty-six guidelines had recommendations for muscle and bone strength. Eight guidelines recommended children and adolescents engage in muscle and bone strengthening activity at least three times per week (Argentina [31], Mexico [34], Netherlands [52], Nordic [25], Paraguay [36], Singapore [45], South Africa [26], WHO [18]) and seven recommended at least 3 days per week (Australia [32], Canada [29], China [50], New Zealand [35], Spain [37], United Kingdom [47], United States [48]). Ghana recommended bone strengthening activity on three or more days per week (Ghana) [44]. The remaining countries used slight variations in the wording of the recommendations (refer to Table 2) with several advising that children over the age of 12 years should incorporate strength activities (Qatar [39], France [53], Philippines [51], Uruguay [49], Turkey [38].

#### Bouts of physical activity

Seven guidelines referred to bouts of physical activity. Two guidelines mentioned bouts of ‘several sessions throughout the day (e.g., 2 bouts of 30 min) (Paraguay [36], Turkey [38]); two suggested several bouts of aerobic activity/brisk exercise of at least 10 min duration (Chile [42], Finland [54]) (Table 2); one recommended three sessions of at least 20 min of “high intensity” physical activity on non-consecutive days (France [43]); and another indicated activities could be performed in multiple shorter periods throughout the day (Mexico [34]). The Philippine guideline [51] was ambiguous recommending ‘at least 20 min of sustained MVPA continuously for a minimum of 30 mins or accumulated bouts of 10 min or longer for children aged 13 to 20 years’.

#### Sedentary and screen time

Seventeen countries mentioned the need to reduce sedentary time. The wording of recommendations for sedentary time varied (refer to Table 2). Ten countries advised limiting sitting/sedentary time for extended/long periods (Australia [32], Canada [29], China [50], Netherlands [52], New Zealand [35], Nordic [25], Spain [37], Switzerland [46], Turkey [38], United Kingdom [47]). Two countries used specific time periods; Austria recommended ‘avoiding long periods of inertia, punctuate periods lasting two or more hours with active stints of physical activity’ [41] and Finland advised ‘not to sit still continuously for longer than one hour’ (Finland) [54].

Eleven countries made specific reference to screen time with varied wording in the recommendations (Australia [32], Canada [29], China [50], Finland [54], France [53], Germany [40], New Zealand [35], Qatar [39], Singapore [45], Spain [37], Uruguay [49]). Ten guidelines did not make reference to sedentary/sitting or screen time (Argentina [31], Chile [42], Ghana [44], Malaysia [33], Mexico [34], Paraguay [36], Philippines [51], South Africa [26], US [48], WHO [18]).

#### Guideline quality

The AGREE II appraisal of each country or international physical activity guideline is provided in Table 4. The domain scores were calculated using the AGREE II Instrument calculation. The scores for each of the six domains were as follows: Scores for *Domain 1: Purpose and Scope* ranged from 41.7 to 100 (Mean = 75.3), *Domain 2: Stakeholder Involvement scores* ranged from 5.5 to 88.9 (Mean = 46.8), *Domain 3: Rigour of Development* ranged from 1 to 99 (Mean = 35.5), *Domain 4: Clarity of Presentation* ranged from 27.8 to 100 (Mean = 69.4), *Domain 5: Applicability,* 2.1 to 87.5 (Mean = 28.9), *Domain 6: Editorial Independence* ranged from 0 to 100 (Mean = 21.5).

**Table 3:** Quick guideline summary

Country	Population age		Physical activity recommendations			Sedentary behaviour recommendations	
	Age group (5–17 years)	Other age groupings	MVPA: at least 60 min/day	Inclusion of vigorous physical activity	Inclusion of bouts of aerobic activity	Inclusion of a sedentary behaviour or sitting	Inclusion of screen time
Argentina	+		+	+			
Australia	+		+	+		+	+
Austria		+	+	+		+	
Canada	+		+	+		+	+
Chile		+	+		+		
China		+	+	+		+	+
Finland		+	+		+	+	+
France		+	+		+	+	+
Germany		+	+			+	+
Ghana		+		+			
Malaysia	+		+	+			
Mexico	+		+	+	+		
Netherlands		+	+	+		+	
New Zealand	+		+	+		+	+
Nordic		+	+	+		+	
Paraguay	+		+	+	+		
Philippines		+	+	+	+		
Qatar	+		+	+		+	+
Singapore		+	+	+		+	+
South Africa	+		+				
Spain	+		+	+		+	+
Switzerland		+	+			+	
Turkey	+		+	+	+	+	
United Kingdom		+	+	+		+	
United States		+	+	+			
Uruguay		+	+			+	+
World Health Organisation	+		+	+			

+ indicates the guideline includes the descriptor/recommendation at the top of the column. ‘Other age groupings’ refers to ages other than 5 to 17 years

**Table 4:** AGREE II Assessment summary

Country	Domain1	Domain2	Domain3	Domain4	Domain5	Domain 6	Total
Argentina	91.7	52.8	21.9	77.8	27.1	0.0	49.7
Australia	100.0	75.0	88.5	94.4	22.9	50.0	78.0
Austria	77.8	41.7	32.3	69.4	27.1	0.0	49.1
Canada	100.0	88.9	99.0	97.2	81.3	100.0	95.3
Chile	58.3	52.8	12.5	66.7	12.5	0.0	39.8
China	86.1	69.4	39.6	69.4	37.5	91.7	63.7
Finland	97.2	30.6	20.8	75.0	37.5	4.2	49.1
France	63.9	44.4	22.9	55.6	6.3	12.5	41.3
Germany	86.1	72.2	60.4	88.9	50.0	0.0	67.4
Ghana	41.7	5.6	5.2	27.8	4.2	12.5	25.8
Malaysia	86.1	33.3	1.0	63.9	16.7	0.0	37.6
Mexico	50.0	30.6	7.3	50.0	2.1	0.0	31.4
Netherlands	100.0	38.9	90.6	77.8	31.3	66.7	75.2
New Zealand	88.9	44.4	81.3	69.4	37.5	50.0	70.5
Nordic	58.3	25.0	14.6	38.9	2.1	8.3	33.2
Paraguay	47.2	27.8	12.5	77.8	14.6	0.0	37.3
Philippines	58.3	38.9	4.2	47.2	6.3	0.0	32.6
Qatar	55.6	44.4	8.3	75.0	22.9	0.0	39.8
Singapore	77.8	52.8	24.0	83.3	18.8	0.0	48.1
South Africa	41.7	22.2	21.9	33.3	29.2	0.0	36.0
Spain	69.4	30.6	5.2	72.2	14.6	8.3	37.9
Switzerland	66.7	25.0	28.1	61.1	35.4	8.3	45.7
Turkey	63.9	30.6	9.4	80.6	20.8	0.0	39.8
United Kingdom	97.2	72.2	52.1	69.4	50.0	4.2	64.3
United States	97.2	75.0	85.4	97.2	70.8	50.0	84.2
Uruguay	77.8	50.0	17.7	55.6	12.5	0.0	41.9
World Health Organisation	94.4	88.9	91.7	100.0	87.5	95.8	93.5
Mean	75.3	46.8	35.5	69.4	28.9	20.8	52.1
Range	41.7 to 100	5.5 to 88.9	1 to 99	27.8 to 100	2.1 to 87.5	0 to 100	25.8 to 95.3

Scores are presented as percentages

## Discussion

National and international physical activity and sedentary behaviour guidelines serve as important tools for health professionals, policy makers, researchers, teachers, parents and children/adolescents. As knowledge of the determinants of physical activity and sedentary behavior among children and adolescents increases, along with a rapid expansion of the evidence base pertaining to the health benefits of different types and duration of physical activities, regular revision and updating of relevant guidelines is essential. This review provides a summary of national and international physical activity and sedentary behaviour guidelines for children and youth a comprehensive summation and insight into guideline quality and variability, whilst highlighting the importance of cross-country comparisons for epidemiological purposes.

While it is acknowledged this review may have been limited by the ability to search in different languages, it is likely that there is still a majority of countries without physical activity and sedentary behaviour guidelines governing and guiding policy and practice. Alternatively, there may be countries who adopt the WHO guidelines without specifically stating it. Guidelines are designed to provide recent evidence-based information that aligns with the recommendation to encourage healthy behaviour [55]. With the increasing burden of the non-communicable disease impacting low, middle and high income countries, reducing risk factors by improving healthy lifestyles is key in the management of this global challenge.

The WHO advocates for multi-sectoral approaches to address declining levels of physical activity, urging governments to develop policies which support interventions to increase physical activity and reduce sedentary behaviour [55, 56]. The current WHO physical activity and sedentary behaviour guidelines are 9 years old, with current plans to update these guidelines in place [18]. As a global leader in the promotion of public health, the WHO provides policies and recommendations that are particularly pertinent for low- and middle-income countries that may not have the resources to appropriately develop or revise physical activity and sedentary behaviour guidelines. The WHO advocates for “scientifically-informed recommendations with a global scope on the benefits, type, amount, frequency, intensity, duration and total amount of physical activity necessary for health benefits” [18]. With a growing body of evidence in the sector it is imperative to update these guidelines on a regular basis and where possible to develop culturally adapted guidelines.

Updating guidelines is also essential in a climate of rapid technological change. The findings from this review found several guidelines were written between eight and 10 years ago [18, 44, 45, 47, 51, 54]. The changes in technology during this timeframe reflect a number of new barriers impacting children’s ability to meet physical activity and sedentary behaviour guidelines. The availability of technologies such as smartphones, laptops, tablets, and gaming consoles as a normal commodity for children has made restricting screen time a difficult task resulting in increased sedentary screen time while impacting opportunities for activity. As indicated in the AGREE II assessment (domain 3 question 14), when guidelines are implemented it is important to include a plan for future review and update [10]. Only four national/international guidelines included a plan to review and update guidelines [18, 29, 32, 52].

There was considerable variability in the age specifications of national guidelines for children and adolescents, which may be the result of cultural differences in formal schooling. Physical activity participation and engagement varies over the life course, there is a notable decline in physical activity as children transition from childhood to adolescence [57]. Some national child and adolescent physical activity and sedentary behaviour guidelines described age groupings which included pre-schoolers and adults [42, 51]. Guideline age groupings should aim to accurately reflect developmental periods to provide appropriate recommendations for the age they are targeting. In some instances age groupings overlapped creating ambiguity in their application (e.g. The Netherlands: 0 to 4 years and 4 to 18 years - and Australia: 0 to 5 years and 5 to 17 years with the distinction that children who were not at school should follow the early years guideline and those at school should follow the 5–17 year guideline) [32, 52]. Further, small differences in guideline wording impact cross-country comparisons. Five countries used categories rather than age ranges with terms such as ‘school aged children’ (Switzerland, Austria) and ‘children and adolescents’ (Nordic, Ghana) and ‘5 years to pre-pubertal and adolescents’ (Uruguay). Subjective categories may lack clarity required by end-users and make cross country comparisons more difficult. Further, terms such as ‘young people’ or ‘youth’ do not accurately reflect the age grouping of child and adolescent guidelines. Youth are defined by the WHO and the United Nations as “individuals in the age grouping 15 to 24-year olds” [58, 59]. There is more ambiguity associated with the terminology ‘young people’: the United Nations Educational Scientific and Cultural Organization (UNESCO) uses the terms ‘young people’ and ‘youth’ interchangeably referring to “individuals aged between 15 and 24 years of age” [60]. The Australian Institute of Health and Welfare refers to young people aged 12 to 17 years and young people aged 15 to 24 years [61]. Regardless, these definitions indicate that there is potential for terminology to inaccurately target the correct age grouping for the guidelines.

The parameter with the most consensus across the guidelines was the recommended time spent in physical activity per day. Nineteen guidelines recommended a minimum of 60 min of MVPA each day. Only one country (Germany) recommended 90 min or more of MVPA every day. However, there were slight variations in the wording of recommendations that can affect the interpretation of the guideline. Four countries recommended 60 min per day, and end users may interpret this as the required amount of time for health benefits without considering any added gains from additional time spent in MVPA [62]. Even small variations in wording could result in misinterpretation. For instance some countries indicated VPA should be incorporated 3 days per week, while others say at least three times per week, which may be confusing for stakeholders. Potentially a child could fulfil the vigorous physical activity guidelines in 1 day if they were to follow the guideline wording ‘three times per week’.

The Nordic countries [25] and South Africa [26] embedded their physical activity guidelines into the national nutrition/dietary guidelines. Estonia [27] combined the nutrition and physical activity guidelines. Whilst these guidelines were comprehensive, it is possible that physical activity guidelines may become lost in nutrition/dietary guidelines, with potentially less opportunity to rigorously review physical activity evidence in its development. Similarly, some countries (Germany [40], Netherlands [52], Philippines [51]) developed a document that included physical activity guidelines across the age spectrum. Whilst these documents were thorough, it is possible that child and adolescent studies to inform the review may have been missed if separate, rigorous search strategies were not conducted, potentially affecting the robustness of the recommendations. These guideline development panels may be limited by a lack of child/adolescent physical activity experts.

There is growing evidence supporting the health impact of regular physical activity in children and adolescents [1, 43]. There is also a growing body of research linking sedentary behaviour and poor health outcomes [2]. The inclusion of sedentary time in guideline development is crucial as children currently spend between 50 and 60% of their day sedentary often replacing physical activity with sedentary, time [2]. Most countries now recognise the health impact of sedentary time on children’s health outcomes, reflected by the inclusion of sedentary behaviour recommendations in 22 of the 29 national and international guidelines. In a recent review, higher levels of screen time were associated with poorer health outcomes with a gradient effect, however the evidence for sedentary behaviour was not consistent [2, 63]. With this in mind and considering the rapid growth in the technology sector (hand held devices, TV, computer, gaming platforms) it is important that guidelines make recommendations to direct stakeholders regarding screen time. However the variability in current sedentary behaviour guidelines reflect the infancy of current evidence to provide a more exact position on the recommended amount of time spent sedentary.

More recently, the potential importance of health opportunities across the entire day have resulted in the implementation of 24-h movement guidelines, with Canada implementing the first 24-h movement guidelines for children and adolescents [12]. The Canadian guidelines combine recommendations for physical activity, sedentary behaviour and sleep for a 24-h period rather than a set of segregated guidelines [12]. Several countries have followed this trend towards 24-h movement guidelines, with New Zealand adopting the Canadian guidelines and Australia ‘adoloping’ the guidelines [64]. Importantly future systematic reviews of physical activity guidelines should incorporate ‘24-h movement’ and associated variations into the search terms to ensure these guidelines are not omitted.

The AGREE II appraisal of guidelines revealed considerable variation in the quality of physical activity guidelines demonstrated by the ‘overall quality score’ ranging from 25.8 to 95.3% (Mean = 61%). Four of the domain average scores were lower than 50%. Domain 3 (rigour used to synthesize and formulate guidelines) is arguably one of the more important domains when assessing quality of guideline development, yet the scores (range 1.5 to 99%; Mean = 35%) indicate a need for more rigorous evidence based development to ensure guidelines are as evidence based as possible [10]. The diversity in the quality of the guidelines were likely to have been impacted by the year they were developed (as recent iteration of guidelines follow a more rigorous evidence based approach) and the socioeconomic status of country (with poorer countries less likely to have the same funding or expertise to support the development of the guideline). Notably the overall AGREE II score for the WHO guideline development was higher than 90% (and will soon be revised). In instances where countries are not able to provide the same level of quality, it is recommended that the WHO Guidelines be used or the GRADE-ADOLOPMENT approach be followed [64].

In the past 10 years there has been a movement towards more rigorous processes for guideline development [12]. Notably, this review has disclosed considerable between-country variability in guideline quality and development. Scientific legitimation is one of the key factors for guideline implementation [55, 65]. In instances where guidelines provide advice without current research to support the recommendation, it should be acknowledged. Health professionals, researchers, and the public rely on the legitimacy of national/international guidelines as a reference point when encouraging healthy lifestyles.

There are several strengths and limitations of this review. The grey literature search enabled a diverse comparison of guidelines that included those that were not written in English; however there may be subtle changes in language between guidelines as they were written in different languages. As a result of the number of guidelines and the diversity in the language of the guidelines only two assessors conducted the AGREE II quality assessment on each of the guidelines. While it is acceptable to have two assessors conduct the AGREE II assessment, it is preferable for up to four assessors to conduct this assessment [10]. Further it was not feasible to have the same assessors conduct the quality assessment of all the guidelines due to the language variation. Further, potentially some guidelines were not captured in this review, as it was not possible to include search terms in all languages. It is also possible that some countries have screen-related guidelines that are separate to their physical activity guidelines, and these may not have been captured in this review.

## Conclusion

There is growing global interest in physical activity and sedentary behaviour guideline development. More recently some countries have included sleep in their guidelines focusing on movement behaviours during a 24 h period.. The findings from this review indicate extensive variability in the quality of country guidelines. Rigorous guideline development is essential to ensure appropriate guidance for population level initiatives. However, low income countries may not have the resources or expertise for guideline development. It is recommended in these instances that the WHO guidelines be used or the GRADE-ADOLOPMENT approach be followed to adopt, adapt, or develop appropriate guidelines for their context.

## Supplementary information

**Additional file 1.** Data base search results.

**Additional file 2.** Table of results for Targeted Websites.

**Additional file 3.** Results of the Content Expert Survey.

## Data Availability

All data generated during the process of this systematic review are included as supplementary files in this published article.

## Abbreviations

AGREEII: Appraisal of guidelines for research and evaluation ii instrument
CDC: Centre for Disease Control
INAHTA: International Information Network, International Network of Agencies for Health Technology Assessment
MVPA: Moderate to vigorous physical activity
PRISMA: Preferred reporting items for systematic reviews and meta-analysis
UNESCO: United Nations Educational Scientific and Cultural Organization
US: United States
VPA: Vigorous physical activity
WHO: World Health Organization
