# Correction to: Comparing and assessing physical activity guidelines for children and adolescents: a systematic literature review and analysis

**DOI:** 10.1186/s12966-020-00993-w

**Published:** 2020-07-09

**Authors:** Anne-Maree Parrish, Mark S. Tremblay, Stephanie Carson, Sanne L. C. Veldman, Dylan Cliff, Stewart Vella, Kar Hau Chong, Maria Nacher, Borja del Pozo Cruz, Yvonne Ellis, Salome Aubert, Billie Spaven, Mohd Jamil Sameeha, Zhiguang Zhang, Anthony D. Okely

**Affiliations:** 1 Faculty of Social Sciences, University of Wollongong, Wollongong, NSW 2521 Australia; 2 Early Start, University of Wollongong, Wollongong, Australia; 3 Illawarra Health and Medical Research Institute, University of Wollongong, Wollongong, Australia; 4 Healthy Active Living and Obesity Research Group, Children’s Hospital of Eastern Ontario Research Institute, Ottawa, Canada; 5 Department of Public and Occupational Health, Amsterdam Public Health Research Institute, Amsterdam University Medical Center, Amsterdam UMC, Vrije Universiteit Amsterdam, Amsterdam, The Netherlands; 6 Motivation and Behavior Program, Institute for Positive Psychology and Education, Faculty of Health Sciences, Australian Catholic University, Sydney, Australia; 7 Nutritional Science Programme, Centre for Community Health, Faculty of Health Sciences, Universiti Kebangsaan Malaysia, Kuala Lumpur, Malaysia

**Correction to: Int J Behav Nutr Phys Act (2020) 17:16**

**https://doi.org/10.1186/s12966-020-0914-2**

Following publication of the original article [1], the authors reported that the co-author’s first name was misspelled in the original article;

1. Incorrect name:

Zhuiguang Zhang

2. Correct name:

Zhiguang Zhang

The original article has been corrected.
