# Supplementary material for: Comparing and assessing physical activity guidelines for children and adolescents: a systematic literature review and analysis
Source: Int J Behav Nutr Phys Act. 2020 Feb 10;17:16. doi: 10.1186/s12966-020-0914-2 (PMC7011603; doi:10.1186/s12966-020-0914-2)
Supplement: Supplementary file 1 — Additional file 1. Data base search results. [file 12966_2020_914_MOESM1_ESM.docx]

**Supplementary File 1:**

| **DATABASE**  **Search** | **YOUTH/ADOLESCENT** | # Results | ✓ |
| --- | --- | --- | --- |
| **PUBMED** | (("physical activity"[Title/Abstract] OR exercise[Title/Abstract]) AND (guideline*[Title/Abstract] OR recommendation*[Title/Abstract]) AND (child*[Title/Abstract] OR youth[Title/Abstract] OR adolescen*[Title/Abstract] OR "school age*"[Title/Abstract] OR "young pe*"[Title/Abstract])) | 1885 | BS  18/7/17 |
| **PROQUEST**  **Central** | ab(("physical activity" OR exercise) AND (guideline* OR recommendation*) AND (child* OR youth OR adolescen* OR "school aged" OR "young pe")) | 2020 | BS 20/7/17 |
| **CINAHL**  **Plus with Full Text** | AB ( "physical activity* OR exercise ) AND AB ( guideline* OR recommendation* ) AND AB ( child* OR youth OR adolescen* OR "school aged" OR "young pe*" ) | 809 | BS 18/7/17 |

Filter: After and including 01/01/2010, Terms in Abstract

| **SEARCH**  **engine** | **YOUTH/ADOLESCENT** | # Results | ✓ |
| --- | --- | --- | --- |
| **GOOGLE SCHOLAR** | ("physical activity" OR exercise) AND (guideline* OR recommendation*) AND (child* OR youth OR adolescen* OR "school age*" OR "young pe*")  *Limit to and including 2010, no citation, no patent, sort by relevance | Approx. ~17,000 | BS  (first 150)  20/7/17 |
| **GOOGLE** | ("physical activity" OR exercise) AND (guidelines OR recommendations) AND (youth OR adolescence OR "school age" OR "young people") AND (site:.gov OR site:.edu OR site:.org)  *Limit to and including 2010, did not include child to make results more specific | Approx. ~1,340,000 | BS  (first 150)  25/7/17  3 pages did not open One website had 5 extra links so total 152 |
