# Supplementary material for: Comparing and assessing physical activity guidelines for children and adolescents: a systematic literature review and analysis
Source: Int J Behav Nutr Phys Act. 2020 Feb 10;17:16. doi: 10.1186/s12966-020-0914-2 (PMC7011603; doi:10.1186/s12966-020-0914-2)
Supplement: Supplementary file 2 — Additional file 2. Table of results for Targeted Websites. [file 12966_2020_914_MOESM2_ESM.docx]

**Supplementary Table 2.**

| **#** | **Targeted Website** | **Search strategy** | **Results** | **Potentialy Relevant results** | **URL** | **Date** |
| --- | --- | --- | --- | --- | --- | --- |
| 1 | World Health Organisation | Physical activity guidelines children | 1 | 1 | http://apps.who.int/iris/bitstream/10665/44399/1/9789241599979_eng.pdf | 18/7/17 |
| 2 | EUROSCAN | Physical activity  guidelines | 0 | 0 |  | 18/7/17 |
|  |  | Physical activity recommendations | 0 | 0 |  |  |
|  |  | Exercise recommendations | 0 | 0 |  |  |
|  |  | Exercise guidelines | 0 | 0 |  |  |
| 3 | INAHTA | Physical activity  guidelines | 0 | 0 |  | 18/7/17 |
|  |  | Physical activity recommendations | 0 | 0 |  |  |
|  |  | Exercise recommendations | 0 | 0 |  |  |
|  |  | Exercise guidelines | 1 | 0 | http://www.inahta.org/members/hct-nhsrc/ |  |
| 4 | Psychextra | CANNOT ACCESS |  |  |  |  |
| 5 | OpenGrey | Physical activity guidelines youth | 3 | 1 | http://www.opengrey.eu/item/display/10068/969970 | 18/7/17 |
|  |  | Physical activity guidelines child | 0 |  |  |  |
|  |  | Physical activity guidelines adolescence | 2 | 1 | http://www.opengrey.eu/item/display/10068/974525 |  |
|  |  | Physical activity guidelines school aged | 0 |  |  |  |
|  |  | Physical activity guidelines young person | 0 |  |  |  |
| 6 | WorldWideScience | (child* OR youth OR adolescen* OR "school aged" OR "young pe*") AND (guideline* OR recommendation*) AND ("physical activity" OR exercise) / From: 2010 | 2204 | 246 | Refined results by choosing >> Topic: Child  Only imported from Papers and Public Access (NOT Multimedia and Data) | 18/7/17 |

*Rationale for 1-3 (CADTH 2015 Grey Matters p.6)
