# Supplementary material for: Comparing and assessing physical activity guidelines for children and adolescents: a systematic literature review and analysis
Source: Int J Behav Nutr Phys Act. 2020 Feb 10;17:16. doi: 10.1186/s12966-020-0914-2 (PMC7011603; doi:10.1186/s12966-020-0914-2)
Supplement: Supplementary file 3 — Additional file 3. Results of the Content Expert Survey. [file 12966_2020_914_MOESM3_ESM.docx]

**Supplementary File 3. Content Expert Survey**

| **What country Report Card did you lead?** | **What guidelines did you follow to determine the proportion of school-aged children and youth meeting guidelines for the Overall Physical Activity indicator?** | **What guidelines did you follow to determine the proportion of school-aged children and youth meeting guidelines for the Overall Physical Activity indicator? [other]** |
| --- | --- | --- |
| Australia | Our own national guidelines (please provide reference or link) | <http://www.health.gov.au/internet/main/publishing.nsf/content/health-pubhlth-strateg-phys-act-guidelines#apa512> |
| Australia | Our own national guidelines (please provide reference or link) | <http://www.health.gov.au/internet/main/publishing.nsf/content/health-pubhlth-strateg-phys-act-guidelines> |
| Belgium | Other (please provide reference or link) | WHO Global recommendations on physical activity for health (<http://www.who.int/dietphysicalactivity/factsheet_recommendations/en/>) |
| Brazil | Other (please provide reference or link) | <http://apps.who.int/iris/bitstream/10665/44399/1/9789241599979_eng.pdf> |
| Canada | Our own national guidelines (please provide reference or link) | <http://www.csep.ca/en/guidelines/24-hour-movement-guidelines> |
| Chile | Other (please provide reference or link) | WHO Guidelines (60 minutes per day MVPA) |
| Colombia | Other (please provide reference or link) | WHO guidelines |
| Denmark | Our own national guidelines (please provide reference or link) |  |
| England | Our own national guidelines (please provide reference or link) | Department of Health. Start active, stay active: a report on physical  activity  for  health  from   the  four  home  countries’  Chief  Medical   Officers.  London,  UK:  Department  of  Health;  2011 |
| Estonia | Other (please provide reference or link) | WHO |
| Finland | Other (please provide reference or link) | At least 60 min MVPA daily, based on WHO recommendation (= minimum recommendation in Finnish recommendations as well) |
| Ghana | Other (please provide reference or link) | Active Healthy Kids Canada guidelines |
| Hong Kong | Our own national guidelines (please provide reference or link) | <http://www.change4health.gov.hk/en/physical_activity/guidelines/youth/index.html> |
| India | Other (please provide reference or link) | WHO Guidelines |
| Ireland | Our own national guidelines (please provide reference or link) |  |
| Japan |  |  |
| Kenya | Other (please provide reference or link) | WHO guidelines |
| Malaysia | Our own national guidelines (please provide reference or link) | based on Global School Health Survey (WHO) |
| Mexico | Other (please provide reference or link) | We don´t have a link, our responses are based on the information and the general agreement in our reunions |
| Mozambique | Other (please provide reference or link) | Canadian |
| New Zealand | Our own national guidelines (please provide reference or link) | <http://www.health.govt.nz/your-health/healthy-living/food-and-physical-activity/physical-activity/how-much-activity-recommended> |
| Nigeria | Other (please provide reference or link) | Guidelines of Canada's Report Card on Physical Activity for Children and Youth 2013. [www.activehealthykids.ca/ReportCard/ReportCardOverview.aspx](http://www.activehealthykids.ca/ReportCard/ReportCardOverview.aspx) |
| Poland | Other (please provide reference or link) | Health Behaviour in-school-aged children (HBSC) |
| Portugal | Other (please provide reference or link) | World Health Organization. (2010). Global Recommendations on Physical Activity for Health. Geneva, Switzerland. |
| Qatar | Our own national guidelines (please provide reference or link) | <http://www.namat.qa/NamatImages/Publications/75/QATAR%20PA%20GUIDLINE%20ENGLISH.PDF> |
| Scotland | Our own national guidelines (please provide reference or link) | Start Active Stay Active 2011 |
| Shanghai, China | Other (please provide reference or link) | At least 1h MVPA daily by WHO |
| Slovenia | Other (please provide reference or link) | WHO Guidelines |
| South Africa | Other (please provide reference or link) | Primarily the WHO Global Recommendations and CDC guidelines for physical activity for youth |
| South Korea | Our own national guidelines (please provide reference or link) |  |
| Spain | Our own national guidelines (please provide reference or link) | <http://www.estilosdevidasaludable.msssi.gob.es/actividadFisica/actividad/recomendaciones/home.htm> |
| Sweden | Our own national guidelines (please provide reference or link) | Nordic Council of Ministers Nordic nutrient recommendations. Copenhagen, Denmark; 2012. Based on WHO guidelines. |
| Thailand | Other (please provide reference or link) | WHO 60 min MVPA daily |
| the Netherlands | Our own national guidelines (please provide reference or link) | Dutch physical activity guidelines (nederlandse norm gezond bewegen) ; <https://www.volksgezondheidenzorg.info/sport/kernindicatoren/beweeg-en-zitgedrag#definitie--node-normen-en-adviezen-voor-sport-en-bewegen> |
| United Arab Emirates | Other (please provide reference or link) | WHO/CDC global guidelines - Current PA guidelines for children were defined as physically active on all seven days for a total of at least 60 minutes per day during the past week |
| US | Our own national guidelines (please provide reference or link) | 2008 Physical Activity Guidelines for Americans |
| Venezuela | Our own national guidelines (please provide reference or link) | [www.inn.gob.ve/](http://www.inn.gob.ve/) |
| Wales | Our own national guidelines (please provide reference or link) | <https://www.gov.uk/government/publications/uk-physical-activity-guidelines> |
| Zimbabwe | Other (please provide reference or link) | The World Health Organization's 60 minutes of MVPA per day |
